# The so-called "Spanish model" - Tobacco industry strategies and its impact in Europe and Latin America

**DOI:** 10.1186/1471-2458-11-907

**Published:** 2011-12-07

**Authors:** Nick K Schneider, Ernesto M Sebrié, Esteve Fernández

**Affiliations:** 1 German Cancer Research Center, Unit Cancer Prevention and WHO Collaborating Centre for Tobacco Control, Im Neuenheimer Feld 280, D-69120 Heidelberg, Germany; 2 Department of Health Behavior, Roswell Park Cancer Institute, Buffalo, New York, USA; 3 Tobacco Control Unit, Cancer Prevention & Control Programme, Institut Català d'Oncologia-IDIBELL, L'Hospitalet de Llobregat, Barcelona, Spain; 4 Department of Clinical Sciences, School of Medicine, Campus de Bellvitge, Universitat de Barcelona, Barcelona, Spain

## Abstract

**Background:**

To demonstrate the tobacco industry rationale behind the "Spanish model" on non-smokers' protection in hospitality venues and the impact it had on some European and Latin American countries between 2006 and 2011.

**Methods:**

Tobacco industry documents research triangulated against news and media reports.

**Results:**

As an alternative to the successful implementation of 100% smoke-free policies, several European and Latin American countries introduced partial smoking bans based on the so-called "Spanish model", a legal framework widely advocated by parts of the hospitality industry with striking similarities to "accommodation programmes" promoted by the tobacco industry in the late 1990s. These developments started with the implementation of the Spanish tobacco control law (Ley 28/2005) in 2006 and have increased since then.

**Conclusion:**

The Spanish experience demonstrates that partial smoking bans often resemble tobacco industry strategies and are used to spread a failed approach on international level. Researchers, advocates and policy makers should be aware of this ineffective policy.

## Background

The World Health Organization Framework Convention on Tobacco Control (WHO FCTC), which was ratified by 174 parties (as of September 2011) including Spain, calls inter alia for the implementation of comprehensive smokefree policies [1]. In January 2006 the Spanish government enacted its former "tobacco control law" (Ley 28/2005), which implemented many measures covered by the FCTC (restrictions on tobacco sales, comprehensive advertising bans, smoking bans at workplaces, etc.), but also allowed owners of "small" hospitality venues (smaller than 100 m^2^) to choose whether smoking should be allowed in their venues or not [2]. Between 2006 and 2010, tobacco taxes have risen, overt promotion has decreased, overall exposure to second hand smoke has declined, and access to cessation advice and services has increased. *Muggli et al*. thoroughly described the genesis of this legislation and *Granero et al*. showed the involvement of different actors, especially those favouring Tobacco Industry interests [3,4]. In this case study we restrict our focus to the so-called "Spanish model" of non-smokers' protection in hospitality venues and its similarities to Philip Morris' (PM) accommodation programmes and demonstrate that thereafter (2006-2011) some European and Latin American countries also introduced similar ineffective legislation.

## Methods

Between October 2009 and February 2010 we searched previously secret internal tobacco industry documents available through litigation in the United States in the electronic Legacy Tobacco Documents Library at the University of California at San Francisco (http://legacy.library.ucsf.edu/)[5]. We used the search terms "Spain", "accommodation", "courtesy of choice" and "traditional hospitality" and triangulated them against online articles of major newspapers using Google (http://www.google.com) and tobacco industry publications using the online database of the Tobacco Journal International (http://www.tobaccojournal.com). In total 3.950 documents included the words "Spain" and "accommodation". After screening the documents the search could be narrowed down to specific accommodation programmes. The number of documents was thus reduced to 401 by adding the specific search terms "Courtesy of Choice" and "Traditional Hospitality" (see table 1). After screening all documents for relevance and discarding duplicates 101 documents were included in the analysis. The key documents identified are summarized in table 2. No relevant additional documents were identified with a search in Spanish (search terms "España" and "acomodación", "hospitalidad" or "convivencia"). The documents were systematically analyzed using a Boolean search [5]. To explore the impact of the "Spanish model" on other countries the search was conducted using the terms "Spanish model" and "hospitality" in English, Spanish and German. Only articles from online newspapers, hospitality and tobacco industry publications referring to tobacco use in hospitality venues were included. 50 relevant news articles were identified and analysed. In addition the information retrieved was triangulated against the legislation of the countries identified in media articles. The relevant documents that finally contributed to the analysis are cited in the final reference list.

**Table 1:** Search terms and number of identified tobacco industry documents

Search terms used in the Legacy Tobacco Documents Library	Number of documents
Spain AND accommodation	3950

Spain AND accommodation AND Courtesy of Choice	979

Spain AND accommodation AND Courtesy of Choice AND Traditional Hospitality	401

Number of documents after screening for relevance and discarding duplicates	101

**Table 2:** Summary and nature of key documents used in the analysis of the tobacco industry strategies

Document	Author	Date	Contents
**Global strategy**			

Worldwide Strategy and Plan to Accommodate the Diverse Expectations Regarding Smoking in Strategic Public Settings and the Role of Ventilation [10].	Prepared in coordination of: -PM International -PM USA -Corporate Affairs -Worldwide Operations and Technology -Worldwide Regulatory Affairs	February 1998	Internal presentation describing the worldwide strategy and plan behind the accommodations programs, including a situation analysis, definition of a common goal, several objectives, and specific strategies to achieve them.

Accommodation and Smoking restrictions [7]	Worldwide Regulatory Affairs	October 1998	Draft plan for 1999 outlining different strategies to solve the issue of: "*Unreasonable restrictions on the use of our tobacco products in public venues*"^6^. The document highlights the objectives identified, suggested strategies and needed. The strategies include inter alia the following international accommodation programs: "*Courtesy of Choice*", "*Traditional Hospitality" *and "*A Smoking Place*".

International Accommodations Programs [6]	Goldberg H., Vice President Environmental Policy, Worldwide Regulatory Affairs, Philip Morris International	July 1999	Internal report and background document which internally demonstrated that the "*International Accommodation Program is very effective"*^5^. The document presents the logic of accommodation, the structure and costs of "Courtesy of Choice" and "Traditional Hospitality", their status and future plans, as well as the barriers of and alternatives to the programs.

Accommodation Programs - where next? [9]	Goldberg H., Vice President Environmental Policy, Worldwide Regulatory Affairs, Philip Morris International	January 2000	Slides and speaking notes highlighting the growth of the program between 1995 and 2000, including number of participating outlets, countries, costs and regulatory benefits in different markets.

**Implementation in Spain and Latin America**			

Consulting Services Agreement [12]	F. J. Braña for Philip Morris Spain and J. Areitio and J.A. Llorente for J.A. Llorente & O. Cuenca	May 1999	Consulting services agreements between PM Spain and the public relations agency J.A. Llorente & O. Cuenca with regard to the management and coordination of among others the "Traditional Hospitality" programme the "FER" (Federación Española de Restaurantes. Cafeterias y Bares) and local hostelry associations with "ATECYR" (Asociación Técnica Española de Climatización y Refrigeración)

Convivencia en Armonía - New Regional Agency [13]	Jacqueline P.Hasty, Supervisor, Communications, Philip Morris International	December 1999	Inter-office correspondence informing about the selection and engagement of J.A. Llorente & O. Cuenca as the new public relations agency to coordinate the Convivencia en Armonia/Cortesía de Elejir programs in Latin America

## Results

### Tobacco industry's accommodation programmes

Aware of the increasing trend towards smoking restrictions and bans in public venues in the early to mid 1990s, PM introduced an international accommodation campaign as a tactical tool to create goodwill amongst legislators and prevent smoking bans [6]. The strategy was coordinated by the Worldwide Regulatory Affairs department with the objective to "*initiate, develop and assist with the implementation of worldwide programs that encourage self-regulation and recognize the benefits of using ventilation effectively to facilitate reasonable accommodation and preserve the social acceptability of smoking*" [7]. It used a three-tier-approach: 1) "*Courtesy of Choice*", to establish smoking and non-smoking rooms, using ventilation to accomplish separation; 2) "*Traditional Hospitality*", to "*enhance the comfort for all customers in small restaurants, cafes, bars and taverns [...] without separating into smoking and non-smoking areas*."; and 3) "*A Smoking Place*", as a smoking lounge concept for large public spaces, e.g. in Spanish airports [8], to demonstrate the "*compatibility of indoor environmental quality and accommodating smoking*" [7].

Although the programmes were run by the International Hotels & Restaurants' Association (IH&RA) in collaboration with public relations firms and ventilation experts, the oversight, strategic planning and funding came from PM's Worldwide Regulatory Affairs department [6]. Since its rollout in 1995, with 460 sites in 9 countries and costs of roughly US$ 1 million, the international accommodation programme expanded considerably, operating in 8.357 sites in 47 countries and costing US$ 15 million in 1999 [6,9]^9^. The ultimate goal of this global programme was to prevent smoking bans and restrictions by creating a reasonable and viable alternative to smoking bans [6,10]. To maximise the impact on legislators on regional, national and European level, key venues were chosen for the programmes, such as the restaurants of the Bilbao Guggenheim Museum and the Bilbao City Auditorium "Palacio Euskalduna" in Spain, as examples for "Traditional Hospitality" [11], as well as the restaurants of the Belgian and Dutch national Parliaments, the Council of Europe (3 locations), European Court of Human Rights (2 locations) and the European Parliament in Strasbourg, as examples of "Courtesy of Choice" [5].

### Birth of the "Spanish Model"

Spain was involved in the programme since 1996 [10] and was one of only 5 countries to pilot "Traditional Hospitality" in 1999, along with Belgium, the Czech Republic, Italy and the Netherlands [6]. In 1999 the public relations agency J.A. Llorente & O. Cuenca was contracted by PM to run the "Traditional Hospitality" programme in Spain and "Courtesy of Choice" in Latin America [12,13]. According to PM the agency's "*presence in Europe adds value to the program since they will be well positioned to transfer knowledge across the two regions and share the many worthy Latin American initiatives*" [13]. In Spain the agency's role also included "*establishing appropiate relations with the Spanish Restaurant Association,(FER), in order to implement the programme sponsored by the IH&RA, with the "FER" acting as cosponsors*" [13]. PM also commissioned an economic study which suggested that a proposed bill would cause a 7% decline in Spanish restaurant revenues and large declines in hospitality industry profits [5]. Besides extensive work by national and international tobacco control advocates and researchers [3,4], the pro-tobacco lobby not only did succeed in "proving the case" and passing a "reasonable legislation" [6,7], but actually managed to lock measures resembling both accommodation programmes in national law: "Courtesy of Choice" for venues bigger than 100 m^2 ^and "Traditional Hospitality" for smaller venues [2].

### Impact on European and Latin American countries

We identified that since 2006 at least nine European and five Latin American countries introduced similar exceptions based on venue size, either on national or sub-national level (province) (Table 3).

**Table 3:** Size dependent partial smoking bans in Europe and Latin America and respective accommodation programs promoted by the tobacco industry

	Earlier existence of PM's accommodation programmes^4^	Partial smoking bans in hospitality venues (2006-2011)	
Measure		Designation of smoking and non-smoking venues	Separation into smoking and non-smoking rooms

Corresponding accommodation programme		"Traditional Hospitality"	"Courtesy of Choice"

**Europe**			

Spain (2006-2010)	TH, CoC	≤100 m^2 ^(accessible area)	> 100 m^2 ^(accessible area)

Denmark (since 2007)	CoC	≤ 40 m^2^	> 40 m^2^

Austria (since 2008)	-	One room venues with < 50 m^2^ (or 80 m^2 ^if separation legally not feasible)	Venues with 2 or more rooms

German states (Bavaria 2008-10; all other states since 2008)	-	One room venues with < 75 m^2^	Venues with 2 or more rooms

Greece (2009-2010)	CoC	≤ 70 m^2^	> 70 m^2^

Croatia (since 2009)	-	≤ 50 m^2^	> 50 m^2^

Switzerland (since 2010)	-	≤ 80 m^2^ (unless stronger Cantonal bans exist)	> 80 m^2^ (unless stronger Cantonal bans exist)

The Netherlands (since 2010)	TH, CoC	≤ 70 m^2^ (if only operated by owner)	> 70 m^2^ (or if not only owner operated)

Czech Republic (since 2010)	TH, CoC	All venues	Smoking rooms without size specification

**Latin America**			

Chile (since 2006)	CoC	≤100 m^2^	Bars, restaurants & casinos > 100 m^2^

Bolivia (2007)	_	Smoking venues (recreational venues for older than 18 years old)	Smoking rooms without size specification

Province of Buenos Aires, Argentina (2008)	CoC	> 400 m^2^	Smoking rooms without size specification

Mexico (2008)	CoC	_	Smoking rooms without size specification

Nicaragua (2010)	_	_	Smoking rooms without size specification

CoC: Courtesy of Choice

TH: Traditional Hospitality

Following the example of Spain, the exceptions for small venues and the focus on traditional hospitality had far-reaching consequences, especially in European countries with strong traditional values linked to tobacco use. Besides the German speaking countries and Croatia, all identified countries with partial bans were originally part of PM's accommodation programmes [6].

Germany was a special case, as PM deliberately decided not to run their programmes there. It reported that Germany had a well organized industry association and PM's management was "*not prepared to alienate the association*" [6]. In 2007 and 2008 however, after the introduction of smoking bans in several German "Länder" (Federal States), tobacco industry lobbyists, the liberal party and the German hospitality industry association Deutscher Hotel- und Gaststättenverband (DeHoGa) advocated for the so-called "Spanish model" as an alternative to a 100% smokefree policy in one-room venues: "*In Spain they found a solution, which is fair to everyone"*[14]. The Spanish law was used as a proxy for the industry's accommodation programmes. In July 2008 the Federal Constitutional Court concluded that although a complete ban would have been constitutional, "freedom of choice" and "mandatory designation" would provide an alternative to a 100% smokefree law - provided that the venues only have one room, are under 75 m^2 ^in size, do not serve prepared food and are not accessible to minors [15]. The main characteristics of the interim solution proposed by the court included the key elements of the Spanish legislation (Ley 28/2005) and outlined the strategies of the accommodation programme operated by PM. The concept of separating smokers and non-smokers in larger venues ("Courtesy of Choice") and allowing smaller venues to choose if smoking is allowed ("Traditional Hospitality") reached constitutionality and was thereafter locked into the state laws of most German states. Only Bavaria became 100% smokefree after a referendum in 2010. In the Netherlands opponents of comprehensive smoking bans advocated for the "German model". In 2010 the Netherlands lifted an earlier ban for all bars, which did not exceed 70 m^2 ^and were solely operated by the owners without employing additional staff. According to news reports this applied to over 3,000 of around 5,500 Dutch bars [16].

In contrast to most partial bans which introduced restrictions based on venue size, some regulations, such as the national laws in the Czech Republic, Bolivia, Mexico, and Nicaragua, and the Province of Buenos Aires in Argentina, allowed smoking rooms or smoking venues without size specifications, probably only following one of PM's accommodation concepts [7]. Since July 2010 it is enough to place a smoking or non-smoking sticker in any Czech restaurant or bar.

In the meantime some countries strengthened their legislation. In 2009, Mexico introduced strict regulations for the implementation of the national law, specifically to the provisions of the smoking rooms (e.g., negative pressure ventilation, sliding doors, etc.) making very difficult to have them in practice. Greece, which introduced a partial smoking ban in 2009, opted for a comprehensive smoking ban in 2010, but retained exceptions for airport lounges, as proposed in PM's "*A Smoking Place*" concept [7].

The "Spanish model" was also used to challenge existing smoking bans, such as in Uruguay, where shortly after the introduction of a comprehensive national smokefree policy in 2006, a national tobacco company started an advertising campaign with the punchline "*Spain and the freedom to choose*" [17], implying that countries under that model would be more democratic and tolerant.

## Discussion

The "Spanish model" has been used as a counter-model to effective smokefree legislation preventing and challenging the introduction of comprehensive smoking bans in several European and Latin American countries. The introduction of smoking bans in public places is often a gradual process, often reaching hospitality venues at a later stage. Most countries with partial smoking bans already had experiences PM's accommodation programmes and could thus have been more vulnerable to the "Spanish model". From recent policy analyses of the Spanish law and scientific studies on secondhand tobacco smoke exposure in hospitality venues we can conclude that separation by size is a failed approach and should not be introduced in any tobacco control legislation [18-25]. Following an extensive public debate and reports on positive developments in countries with comprehensive smoking bans, such as Ireland and the United Kingdom, and the scientific evaluations of the impact of the law, the Spanish government enacted new legislation in January 2011 amending its law and removing all exceptions for hospitality venues (new Ley 42/2010). Thus the "Spanish model" will no longer be that of a partial and weak ban, but a total one, as recommended by the FCTC [25].

What happened in Spain and elsewhere illustrates how partial bans, as promoted by the tobacco industry and parts of the hospitality sector, do not protect people against second-hand smoke. PM's strategy of using a common public relations agency for the management of the programs in Spain and Latin America demonstrates Spain's strategic position in the communication and dissemination of the programs across both regions [12]. The "new Spanish model" is an example of good practice for those countries aiming to go smoke-free. The Spanish experience should be used to counter the introduction of partial smoking bans across the globe and to support the global implementation of the FCTC. As the old "Spanish model" is dead, tobacco industry and their front groups are expected to use new terms, such as the "German model", to sell their accommodation programmes to policy makers and the media.

## Conclusion

The Spanish experience not only demonstrates that partial bans often resemble tobacco industry strategies and are used to spread a failed approach on international level, but also that they are reversible. Researchers, advocates and policy makers should thus be aware of the origins of partial smoking bans, especially the so-called "Spanish model".

## Competing interests

The authors declare that they have no competing interests.

## Authors' contributions

All three authors were involved in study design, data collection and manuscript development. NKS researched the tobacco industry documents on Spain and Europe and prepared the first manuscript. ES provided the data on Latin America and EF further documentation on Spain. All three authors commented all versions of the manuscript and approved the final version.

## Pre-publication history

The pre-publication history for this paper can be accessed here:

http://www.biomedcentral.com/1471-2458/11/907/prepub
